# Renal angiomyolipoma with bleeding

**DOI:** 10.2349/biij.3.4.e8

**Published:** 2007-10-01

**Authors:** M Muttarak, N Pattamapaspong, B Lojanapiwat, B Chaiwun

**Affiliations:** 1 Department of Radiology, Chiang Mai University, Chiang Mai, Thailand; 2 Department of Surgery, Chiang Mai University, Chiang Mai, Thailand; 3 Department of Pathology, Chiang Mai University, Chiang Mai, Thailand

## HISTORY

A 47-year-old woman was referred to our hospital for the management of her large abdominal mass. She had a history of right flank pain off and on for 2-3 years. She was admitted to the provincial hospital in the previous four days due to right flank pain and nausea. She had no history of trauma. Abdominal computed tomography (CT) was performed and liposarcoma was suspected in the right flank. She was pale and was found to have an ill-defined 14x17 cm mass with mild tenderness in the right flank. Her blood pressure and pulse was 130/80 mm Hg and 80/minute, and body temperature was 36.5^o^C. Laboratory investigations were: hemoglobin 6.2 g/dL, hematocrit 20.3%, white blood cell count 9.6x103/mm^3^ and creatinine 1 mg/dl. Urinalysis revealed red blood cell 1-2 while white blood cell 8-12 in the high power field.

## IMAGING FINDINGS

**Figure 1:**
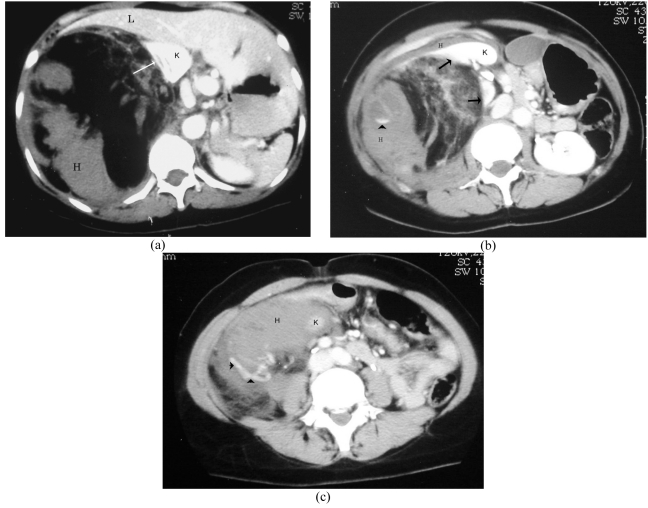
Enhanced-CT images at (A) upper pole (B) middle pole, and (C) lower pole of the right kidney (K) show a 13x15x20 cm fat-containing mass arising from the lateral aspect of the kidney with sharp defect (arrows) in renal parenchyma, enlarged vessels (arrowheads), intratumoural and perinephric hemorrhage (H), and compression of the liver (L).

Abdominal CT showed a large predominantly fat-containing mass arising from the lateral aspect of the right kidney with compression of the liver, enlarged vessels within the mass, intratumoural and perinephric hematoma (Figure 1).

## CLINICAL COURSE

Symptomatic treatment with blood transfusion was given but she still had anemia and flank pain. An elective simple right neprectomy was performed. She made an uneventful recovery.

## PATHOLOGICAL FINDINGS

**Figure 2:**
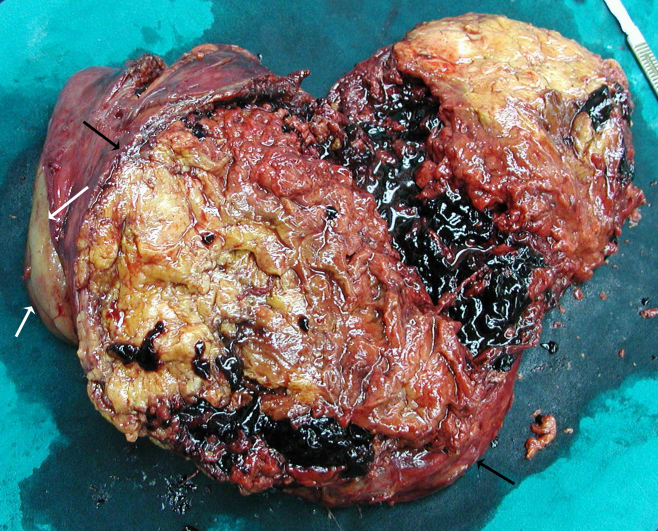
Photograph of an excised specimen reveals a large circumscribed mass (black arrows) with variegated cut surface, showing predominance yellowish areas of fat admixed with intratumour hemorrhage and necrotic foci. The normal renal parenchyma (white arrows) is compressed.

**Figure 3:**
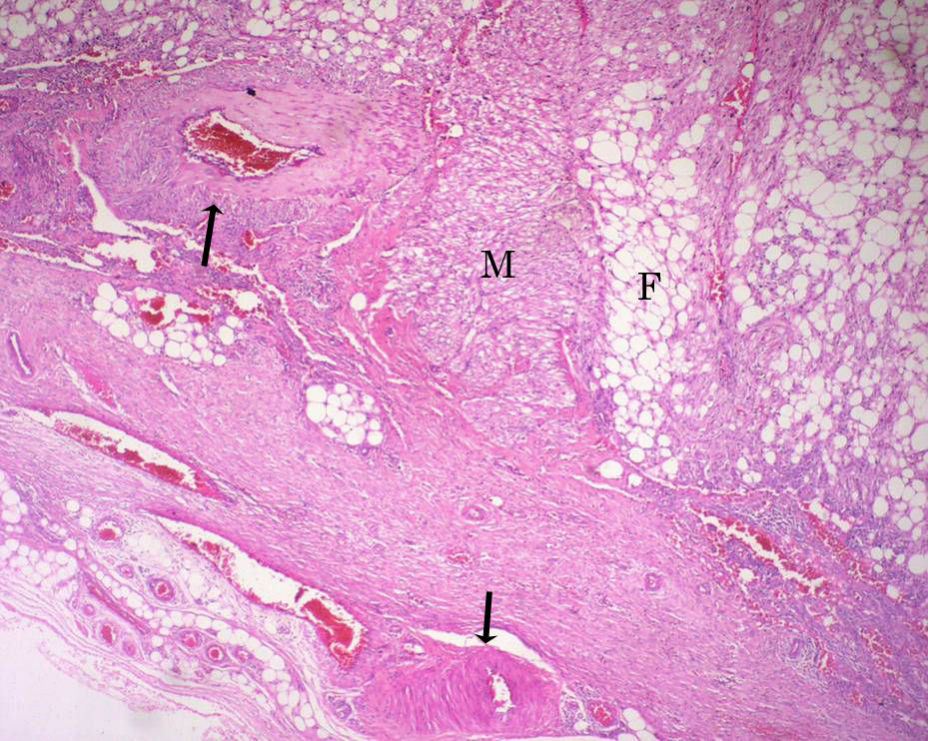
Photomicrograph shows admixture of tortuous thick-walled blood vessels (black arrows), sheets of mature adipose tissue (F) and bundles of smooth muscle fibres (M) (hematoxylin and eosin stain; x40).

On gross examination, there was a large circumscribed fat-containing mass with hemorrhage, and compression of the renal parenchyma (Figure 2). Microscopic examination revealed tortuous thick-walled blood vessels, sheets of mature fat cells and bundles of muscle fibres (Figure 3), consistent with angiomyolipoma (AML).

## DISCUSSION

AMLs are uncommon benign tumours of the kidney composed of varying amounts of fat, smooth muscle and abnormal thick-walled blood vessels. AMLs may occur as isolated lesions or are associated with tuberous sclerosis (TS). Isolated or sporadic AMLs account for 80 to 90% of reported cases and commonly occur in women aged 40 to 70. The lesions in this group are usually unilateral and focal. AMLs associated with TS are usually bilateral and multifocal, and can occur at any age and in either sex. Most patients are asymptomatic and the tumour is often incidentally detected during ultrasonography (US) or CT [1,2]. When symptoms do occur, common presenting symptoms are flank or abdominal pain, palpable mass and hematuria related to spontaneous intramural or extramural hemorrhage. The main complication of AMLs is hemorrhage, which is related to the tumour size, increased vascularity and abnormal thick-walled vessels that are predisposed to the formation of microaneuryms and bleeding. AMLs larger than 4 cm in diameter increase the risk for hemorrhage [3]. Although AMLs are considered a benign lesion, reports of growing lesions, invasion into the inferior vena cava and regional lymph nodes have been noted [1].

**Figure 4:**
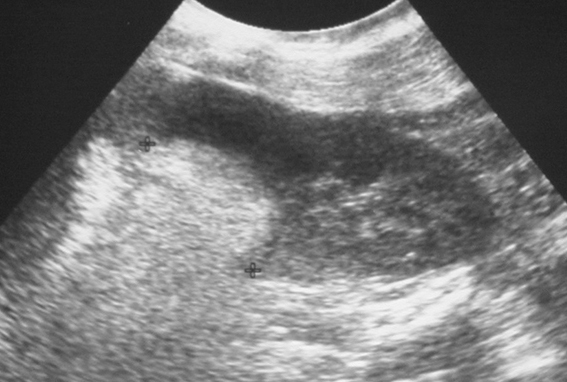
Oblique coronal US scan of the kidney of another patient with uncomplicated AML shows a hyperechoic mass (cursors) in the upper pole.

Preoperative diagnosis of AMLs was difficult in the past and rarely made. Today, with the widespread use of US and CT, more AMLs are diagnosed preoperatively. With US, AMLs appear as a marked hyperechoic mass (Figure 4) due to high fat content and multiple tissue interfaces produced by fat and multiple vessels [2]. However, this high echoic mass is not pathognomonic for AMLs. Other renal tumours including renal cell carcinoma, liposarcoma, atypical Wilms’ tumour, lymphoma, lipoma, oncocytoma, and cavernous hemangioma may be hyperechoic. CT is highly specific to fatty tissue in the lesion and is often performed to confirm the diagnosis [1,2]. Although there have been a few reports of fat occurring in renal cell carcinomas, calcification has also been detected [4]. Whereas, AMLs rarely contain calcification [5], therefore a diagnosis of AMLs should not be made if a lesion contains fat and calcification. Rarely, renal tumours including lipoma, liposarcoma, Wilms’ tumour, teratoma, oncocytoma, and xanthogranulomatous pyelonephritis contain sufficient quantities of fat to be detected at CT [6]. A large exophytic renal angiomyolipoma may be difficult to differentiate from a well-differentiated perirenal liposarcoma because they both contain fat. Careful evaluation for a defect in the renal parenchyma and a presence of enlarged vessels can help differentiate angiomyolipoma from liposarcoma [7].

Some AMLs may contain very small amounts of fat that can be overlooked if they are not carefully evaluated. If a small amount of fat is suspected in a renal mass, unenhanced CT scan with thin sections combined with a small area for attenuation analysis is recommended. A small number of AMLs contain predominantly muscle and blood vessels with scanty fat component. These AMLs appear as non-fatty renal mass and the imaging differentiation from renal cell carcinoma seems impossible.

Treatment of AMLs depends on the size of the lesion. If an isolated AML is less than 4 cm in diameter, conservative follow-up with either US or CT is recommended. If the AML is larger than 4 cm, it has a greater tendency to bleed. Therefore, patients with AMLs greater than 4 cm and bleeding or uncontrollable pain should undergo renal-sparing or renal arterial embolisation if it is available [3]. The case presented in this paper did not have preoperative embolisation because angiography service was not available at that time.

In summary, this case is a large solitary renal AML with spontaneous bleeding. Clinical course, diagnosis and management of renal AMLs are discussed.
